# The Impact of Combined Chemotherapy and Intra-Tumoural Injection of Phosphorus-32 Microparticles on Vascularity in Locally Advanced Pancreatic Carcinoma

**DOI:** 10.3390/cancers16193412

**Published:** 2024-10-08

**Authors:** Amanda Huoy Wen Lim, Joshua Zobel, Madison Bills, William Hsieh, Benjamin Crouch, Rohit Joshi, John-Edwin Thomson, EuLing Neo, Li Lian Kuan, Romina Safaeian, Edmund Tse, Christopher K. Rayner, Andrew Ruszkiewicz, Nimit Singhal, Dylan Bartholomeusz, Nam Quoc Nguyen

**Affiliations:** 1 Department of Gastroenterology and Hepatology, Royal Adelaide Hospital, Port Road, Adelaide, SA 5000, Australia; amanda.lim@sa.gov.au (A.H.W.L.); joshua.zobel@sa.gov.au (J.Z.); romina.safaeian@sa.gov.au (R.S.); edmund.tse@sa.gov.au (E.T.); chris.rayner@adelaide.edu.au (C.K.R.); dylan.bartholomeusz@sa.gov.au (D.B.); 2 School Medicine, The University of Adelaide, Adelaide, SA 5005, Australia; andrew.ruszkiewicz@sa.gov.au (A.R.); nimit.singhal@sa.gov.au (N.S.); 3 Department of Nuclear Medicine, Royal Adelaide Hospital, Adelaide, SA 5000, Australia; madison.bills@sa.gov.au (M.B.); william.hsieh@sa.gov.au (W.H.); benjamin.crouch@sa.gov.au (B.C.); 4 Medical Oncology, Lyell McEwin Hospital, Adelaide, SA 5112, Australia; rohit.joshi@sa.gov.au; 5 Department of Hepatobiliary Surgery, Royal Adelaide Hospital, Adelaide, SA 5000, Australia; john.edwin-thomson@sa.gov.au (J.-E.T.); euling.neo@sa.gov.au (E.N.); 6 Department of Upper Gastrointestinal Surgery, Queen Elizabeth Hospital, Adelaide, SA 5011, Australia; lilian.kuan@sa.gov.au; 7 Surgical Pathology, SA Pathology, Adelaide, SA 5000, Australia; 8 Centre of Cancer Biology, University of South Australia, Adelaide, SA 5000, Australia; 9 Oncology, Royal Adelaide Hospital, Adelaide, SA 5081, Australia

**Keywords:** pancreatic cancer, vascularity, 32 phosphorus microparticles, intra-tumoural radiation, chemotherapy

## Abstract

**Simple Summary:**

Despite the advances in medicine, the 5-year survival rate for pancreatic ductal adenocarcinoma (PDAC) is still dismal and has not improved. New treatment options are required. Some data have been promising for endoscopic ultrasound-guided phosphorus-32 (^32^P) microparticle implantation. Given the role of poor vascularisation in the poor response of pancreatic cancer to standard therapy, such as chemotherapy, we hypothesised that EUS-guided ^32^P microparticle implantation improves chemotherapy delivery to and effects on the primary tumour. No treatment option has been shown to consistently improve tumour vascularity in PDAC, but a treatment that is able to improve vascularity may work synergistically with chemotherapy to improve outcomes in PDAC.

**Abstract:**

Background: Poor intra-tumoural vascularity contributes to a lack of response to chemotherapy in pancreatic cancers. Preliminary data suggest that the addition of endoscopic ultrasound (EUS)-guided intra-tumoural injection of phosphorus-32 (^32^P) microparticles to standard chemotherapy is potentially beneficial in locally advanced pancreatic cancer (LAPC). We aimed to assess changes in pancreatic tumour vascularity following ^32^P implantation, using contrast-enhanced EUS (CE-EUS). Methods: This was a prospective single-centre trial from January 2022 to 2024 of patients with unresectable, non-metastatic LAPC undergoing standard FOLFIRINOX chemotherapy and ^32^P implantation. We performed CE-EUS pre-implantation after two chemotherapy cycles and 4 and 12 weeks after implantation. Time–intensity curves were analysed for 90 s after IV contrast bolus to ascertain peak intensity and intensity gain. Results: A total of 20 patients underwent ^32^P implantation, with 15 completing 12-week follow-up. The technical success of ^32^P implantation was 100%. The median primary tumour size reduced from 32 mm (IQR 27.5–38.75) pre-implantation to 24 mm (IQR 16–26) 12 weeks post-implantation (*p* < 0.001). Five patients (25%) had tumour downstaging, and four underwent resections. The baseline (pre-implantation, post-chemotherapy) median intensity gain of contrast enhancement within the tumour was 32.15 (IQR 18.08–54.35). This increased to 46.85 (IQR 35.05–76.6; *p* = 0.007) and 66.3 (IQR 54.7–76.3; *p* = 0.001) at 4 weeks and 12 weeks post-implantation, respectively. Over a median follow-up of 11.2 months (IQR 7.8–12.8), 15/20 (75%) of patients remained alive, with 3/20 (15%) demonstrating local disease progression. Overall survival was not significantly different between patients with or without an increased intensity of 10 a.u. or more at 12 weeks post-implantation. Conclusion: This is the first clinical study to demonstrate treatment-induced increased vascularity within pancreatic primary tumours, which followed ^32^P implantation and FOLFIRINOX chemotherapy. Larger comparative trials are warranted.

## 1. Introduction

Pancreatic ductal adenocarcinoma (PDAC) is currently the seventh leading cause of cancer death worldwide and is projected to become the second leading cause of cancer death worldwide within the next ten years, carrying a poor prognosis [1,2]. Although resection is the only curative option, 80% of PDACs are unresectable at presentation [2]. Despite the advances in chemotherapy, chemoradiotherapy and immunotherapy, responses in patients with locally advanced pancreatic cancer (LAPC) remain poor due to factors including a dense stroma, immunosuppressive environment, and hypoxia within the primary tumour related to poor vascularity [3,4]. This micro-environment restricts the access of systemic chemotherapy to the primary tumour.

Although the role of intra-tumoural vascularity has been controversial, there is evidence that poor vascularity and hypoxia have an adverse prognosis [5,6]. This has led to the hypothesis that vascular normalisation may lead to improved outcomes, potentially by increasing drug delivery to tumour tissue, a concept that has been supported in animal studies [7,8]. For example, increased microvascular density in a murine PDAC xenograft model resulted in higher chemotherapy delivery and the inhibition of tumour growth [9]. Higher intra-tumoural blood flow has also been associated with improved treatment response in patients with unresectable pancreatic cancer undergoing chemotherapy [7]. Some studies have reported a relationship between increased intra-tumoural microvessel density and metastases; however, this was in the setting of abnormal tumour angiogenesis rather than vascular normalisation [10,11]. Tumour angiogenesis involves the development of more aberrant disorganised vessels, but this is often associated with tumour growth as well as hypoxia, rendering chemotherapy and radiation less effective [12,13].

Therapeutic approaches that normalise tumour vasculature, regulate blood flow and increase perfusion are required. Although angiogenesis inhibitors have been shown to normalise tumour vasculature, the effect is transient, and the continuation of anti-angiogenic treatment can result in further hypoxia and reduced drug delivery to the tumour [14,15]. In PDAC, anti-angiogenic treatments have not been effective [10,16].

An animal model has shown that radiotherapy may reduce intra-tumoural interstitial pressure and, thereby, increase vascularity and drug delivery in cancers. However, chemoradiotherapy has not shown a survival benefit in LAPC compared to chemotherapy alone [17]. Current radiotherapeutic options include conventionally fractionated external beam radiotherapy (EBRT) and stereotactic body radiation therapy (SBRT) regimens, which deliver between 50–60 Gy to the tumour. The injection of phosphorus-32 (^32^P) microparticles into the tumour under endoscopic ultrasound (EUS) guidance represents a novel brachytherapy approach [18]. Beta-radiation-emitting ^32^P microparticles allow a higher dose of radiation (100 Gy; 98% delivered over 81 days) to be administered whilst limiting damage to surrounding organs, including the gastrointestinal tract [18]. It is typically performed in addition to systemic chemotherapy (FOLFIRINOX or gemcitabine + nab-paclitaxel), which is given per standard of care. Combined chemotherapy and ^32^P implantation via EUS has been reported to achieve an 82% local disease control rate and a 20% resection rate in patients with locally advanced pancreatic cancer [19].

The mechanism of achieving local disease control is unclear but may be related to changes to the tumour microenvironment. Contrast-enhanced endoscopic ultrasound (CE-EUS) has been used as a surrogate marker of tumour blood volume and blood flow, with this technique demonstrated to have a good correlation with patterns of vascularity in surgical resections [4,20,21]. This study aimed to use CE-EUS to assess whether combined ^32^P microparticles and chemotherapy can alter pancreatic tumour vascularity as a possible mechanism for its recently demonstrated encouraging results.

## 2. Materials and Methods

### 2.1. Study Design

We performed a prospective single-centre (Royal Adelaide Hospital) study. Consecutive patients from January 2022 to January 2024 discussed at hepato-pancreatico-biliary multidisciplinary meetings at centres within South Australia with unresectable LAPC undergoing FOLFIRINOX first-line chemotherapy were offered enrolment if they met the following criteria: (a) ≥18 years of age at screening; (b) histologically proven adenocarcinoma of the pancreas; (c) unresectable locally advanced pancreatic carcinoma; (d) ECOG of 0–1; (e) standard first-line chemotherapy with folinic acid, fluorouracil, irinotecan and oxaliplatin (FOLFIRINOX) would be/had commenced within 14 days of enrolment; (f) life expectancy of ≥3 months at the time of screening, as judged by the investigators; and (g) platelet count ≥100,000/mm^3^. Patients were excluded if they met the following criteria: (a) had evidence of distant metastases; (b) had more than one primary lesion; (c) had prior radiotherapy for pancreatic cancer; (d) had a known allergy or history of hypersensitivity to silicon or phosphorus; and (e) did not consent to chemotherapy or did not complete the first 2 cycles of chemotherapy before implantation. Patients consented to EUS-guided ^32^P implantation (OncoSil™; OncoSil Medical, Sydney, Australia) being performed after the 2nd or 3rd cycle of chemotherapy. Baseline tumour characteristics, including size, location and histology, were recorded from abdominal computed tomography and diagnostic EUS performed prior to enrolment. Other baseline investigations included (a) blood tests for serum cancer antigen 19-9 (CA 19-9), liver function tests (LFTs), coagulation profile and complete blood examination (CBE) and (b) quality of life (QoL) assessment using the European Organisation for Research and Treatment of Cancer (EORTC) QLQ-C30 questionnaire. This 30-item cancer-specific questionnaire involves a global health status/QoL scale, 5 functioning scales (physical, role, cognitive, emotional and social functioning), 3 symptom scales (fatigue, pain and nausea/vomiting) and 6 single items (dyspnoea, insomnia, appetite loss, constipation, diarrhoea and financial difficulties). All items employ a 4-point Likert scale ranging from 1 (not at all) to 4 (very much), with the exception of the global health status/QoL scale, which uses a 7-point scale [22]. Clinically relevant change was defined as a difference of 10 points or more.

After 2 cycles of FOLFIRINOX (timing dependent on available endoscopic procedural scheduling), patients underwent CE-EUS and EUS-guided ^32^P implantation, followed by a Bremsstrahlung abdomen planar static, followed by single-bed single photon emission computed tomography (SPECT) imaging within 4 h to confirm the intra-tumoural distribution of microparticles. Following implantation, patients continued chemotherapy as scheduled. At 4 weeks and 12 weeks post-implantation, EUS and CE-EUS were repeated to assess changes in tumour vascularity and tumour size and assess gastrointestinal adverse effects. The blood tests and EORTC QLQ-C30 questionnaire were repeated at these time points, and abdomen/pelvis CT was also repeated at 12 weeks, with clinical outcomes defined as per RECIST 1.1 criteria [20]. Progressive disease (PD) was defined as a ≥20% increase in the sum of the longest diameter of target lesions or the emergence of new lesions. The local disease control rate was defined as complete response (CR: the disappearance of lesions), partial response (PR: a 30% decrease in the sum of the longest diameters of target lesions) or stable disease (SD: not meeting criteria for CR, PR or PD). All patients were monitored for acute gastrointestinal (GI) toxicities, including nausea, vomiting, abdominal pain, diarrhoea and gastroduodenal ulcers and bleeding. All toxicities were scored according to the Common Terminology Criteria for Adverse Events (CTCAE), version 5.0. This study protocol was approved by the Central Adelaide Local Health Network Human Research Ethics Committee (HREC/19/CALHN/484) and has been registered on ANZCTR (ACTRN12622000403752).

### 2.2. CE-EUS

All EUS procedures were performed under conscious sedation with Propofol by a single endosonographer (N.Nguyen), using a linear echoendoscope (GF-UCT180, Olympus Medical, Tokyo, Japan) and a Prosound F75 ultrasound processor (Aloka, Tokyo, Japan). The location and size of the pancreatic mass were recorded. Contrast enhancement was undertaken by the intravenous injection of a 0.5 mL bolus of Perflutren lipid microsphere enhancing contrast media (Definity^®^, Lantheus Medical Imaging, Billerica, Massachusetts) followed by a 5 mL saline flush. CE-EUS was subsequently used to scan the pancreatic mass and surrounding parenchymal structures for a minimum of 90 s (s) for the complete observation of the arterial (10–30 s) and venous (30–90 s) phases (a mechanical index of 0.4, a transmitting frequency of 5–13 Hz and a frame rate of 10 images per second). If the enhancement was unsatisfactory, the intravenous bolus and saline flush were repeated.

**Figure 1:**
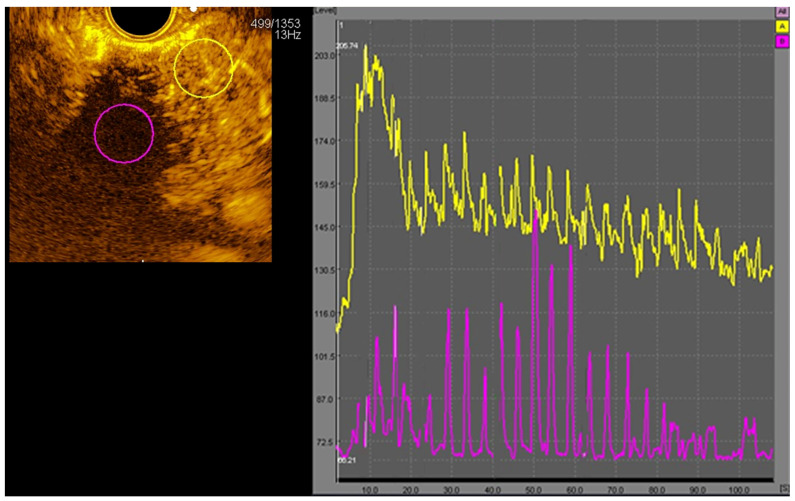
Time–intensity curves for pancreatic tumour (purple ROI) and parenchymal tissue (yellow ROI). ROI—region of interest.

Time–intensity curves were generated using contrast-enhanced analysis quantification software. Two circular regions of interest (ROIs) were selected by consensus with two investigators (A.Lim and N.Nguyen), one from the tumour and the other from normal pancreatic tissue distant from large vessels (Figure 1). Although time–intensity curves were generated from the time of recording contrast images, this did not always coincide with starting at the time of the contrast bolus, and therefore, the time of the contrast bolus was separately recorded. Echo intensity (in arbitrary units [a.u.]) was assessed within the tumour at baseline and during the arterial phase of contrast enhancement (10–30 s after the contrast bolus), and both peak intensity and time to peak after contrast injection were recorded. Hypoenhancement was defined as less intense enhancement than surrounding pancreatic tissue.

### 2.3. ^32^P Implantation

The activity of ^32^P was calculated prior to implantation to deliver 100 Gy to the tumour mass. The required volume (mL) of the ^32^P 6.6 MBq/mL suspension to be dispensed and implanted was calculated as tumour volume (mL) × 8/100 [15]. Implantation was performed using a disposable EUS-FNA 22-gauge needle, loaded through the biopsy channel of the echoendoscope and advanced slowly through the gastric or duodenal wall into the target pancreatic tumour, avoiding vessels and surrounding organs. After ^32^P injection, the needle sheath was pushed out into the gastric lumen, the needle retracted into the sheath and saline flushed into the gastric lumen. The sheath, echoendoscope and needle complex were then removed from the patient and flushed again into a biohazard disposal container. Following implantation, participants were observed for 4 h and discharged after the Bremsstrahlung scan if clinically stable and free of pain.

### 2.4. Statistical Analysis

The primary outcome was a change in tumour vascularity at 4 and 12 weeks post-^32^P implantation. Secondary outcomes included changes in primary tumour size at 12 weeks post-implantation, clinical response as per RECIST criteria v1.1, CA 19-9 levels at 12 weeks post-implantation, downstaging and resection rates, local and distant progression and overall survival. Outcomes were reported as medians (interquartile range) for variables that were not normally distributed and means (standard deviation) if normally distributed. The Kolmogorov–Smirnov test was used to test normality. Paired Wilcoxon signed-rank tests were used to analyse non-parametric paired variables (2 categories), whilst the Friedman test was used for >2 categories. Paired t-tests were used for normally distributed paired variables. Spearman’s rho correlation was used to assess the correlation between two continuous variables that were not normally distributed.

Enrolling 20 participants ensures 80% power to detect a 15% difference in tumour vascularity from ^32^P implantation to 12 weeks post-^32^P implantation (using an initial mean of 47) at the one-tailed 0.05 significance level [21]. Analyses were performed using IBM SPSS version 27 (IBM SPSS Statistics, IBM Corporation, Armonk, NY, USA). *p* < 0.05 was considered statistically significant.

## 3. Results

Twenty patients (45% male, median age 65 years) with unresectable LAPC underwent combined chemotherapy and intra-tumoural ^32^P implantation. Baseline demographics and tumour characteristics are summarised in Table 1.

**Figure 2:**
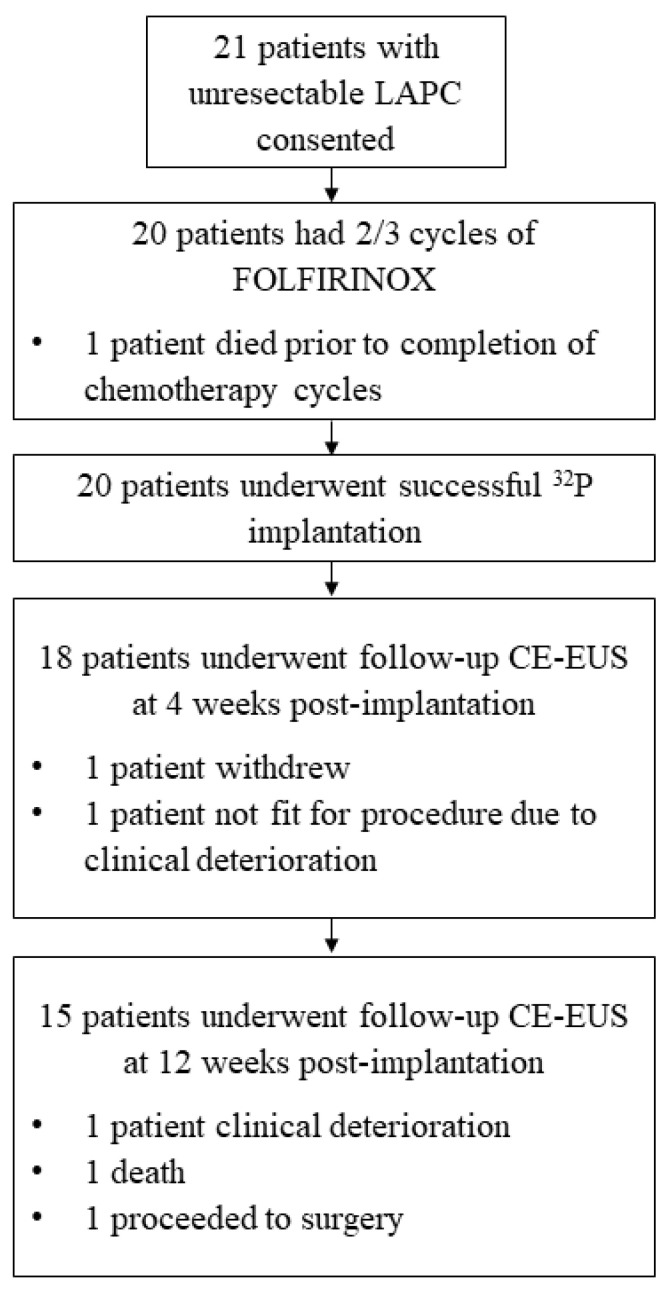
Patient flowchart.

The technical success of ^32^P implantation was 100%, with no procedural complications. The median injected volume of ^32^P was 1.05 mL (IQR 0.98–1.9). Ten patients (50%) had a minimal residual tracer of ^32^P in the small bowel on the Bremsstrahlung scan due to the flushing of the EUS needle within the gastric lumen; ^32^P was confined to the pancreas in all others. None of these patients had any resulting complications. Fifteen patients completed the 12-week follow-up (see Figure 2).

### 3.1. CE-EUS Results

**Figure 3:**
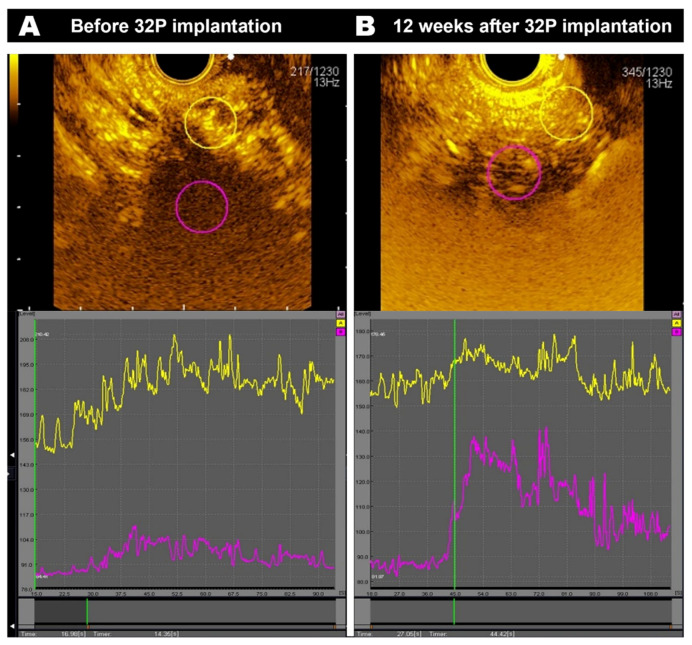
Time–intensity curves in a patient (**A**) pre-^32^P implantation and (**B**) 12 weeks post-implantation. Vertical green lines represent the time of the image on the left. The *Y*-axis represents the intensity (arbitrary units), whilst the *x*-axis is the time (seconds) from video recording (but adjusted for the time of the contrast bolus) at the origin.

**Figure 4:**
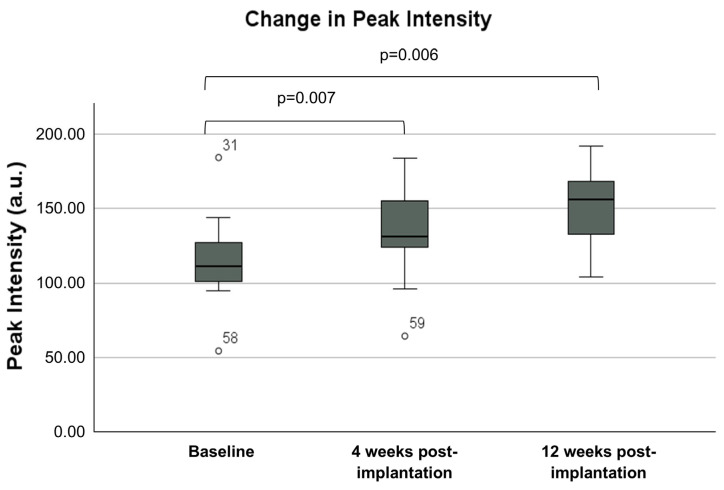
Change in peak intensity. a.u.—arbitrary units.

**Figure 5:**
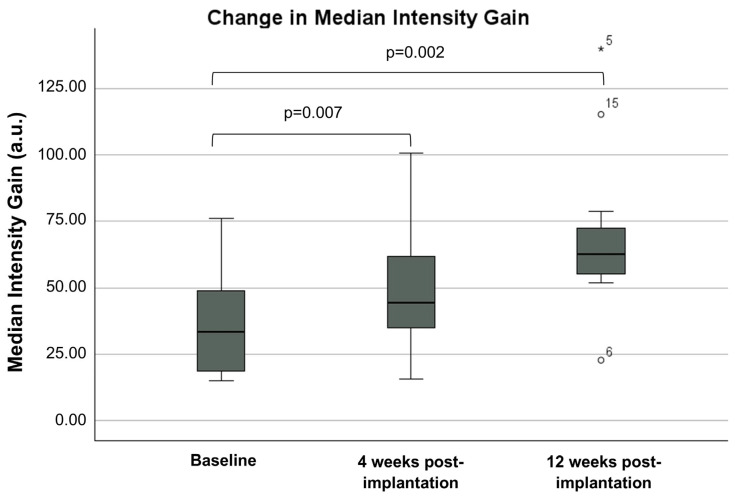
Change in median intensity gain. a.u.—arbitrary units.

All tumours demonstrated hypoenhancement (lower peak enhancement intensity relative to surrounding pancreatic tissue, which is suggestive of hypovascularity) at baseline (median 111.3 a.u. versus 178.1 a.u.; *p* < 0.001) (Figure 3). The median peak intensity of contrast enhancement within the tumour pre-implantation after a median two cycles of chemotherapy was 111.3 a.u. (IQR 99–128.7), which increased to 131.35 a.u. (IQR 122.6–156.5; *p* = 0.007) at 4 weeks and 156 a.u. (IQR 127.0–170; *p* = 0.006) at 12 weeks post-implantation (Figure 4). The median peak intensity of normal pancreatic parenchyma did not change (baseline 178.1 [IQR 161.5–195.4] vs. 4 weeks 182.0 a.u. [IQR 174.2–194.7] vs. 12 weeks 181.7 a.u. [IQR 156.7–212.9]; *p* = 0.584). The median intensity gain of contrast enhancement within the tumour pre-implantation was 32.15 a.u. (IQR 18.08–54.35) and increased to 46.85 a.u. (IQR 35.05–76.6; *p* = 0.007) and 66.3 (IQR 54.7–76.3; *p* = 0.002) at 4 weeks and 12 weeks post-implantation, respectively (Figure 5). An increase in peak intensity was demonstrated in 13/15 patients (86.7%), whilst an increase in intensity gain was demonstrated in 14/15 patients (93.3%).

### 3.2. Clinical Outcomes

The median longest primary tumour size decreased from 32 mm (IQR 27.5–38.75) pre-implantation to 24 mm (IQR 16–26) at 12 weeks post-implantation (*p* < 0.001). Five patients (25%) had tumour downstaging, and four underwent resection (100% R1). One patient refused surgical treatment.

Median CA19-9 fell from 183.5 U/mL (IQR 69.8–274.8) to 85 U/mL (IQR 37–250) at 4 weeks post-implantation (n = 15; *p* = 0.002) and to 43 U/mL (22.3–191) at 12 weeks post-implantation (n = 16; *p* = 0.01).

**Figure 6:**
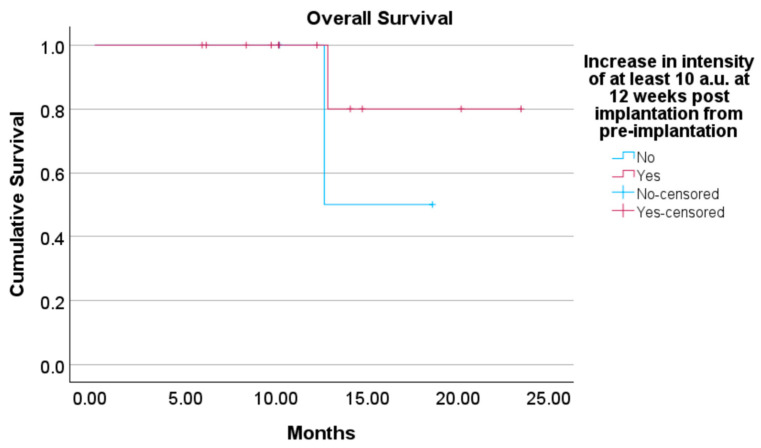
Kaplan–Meier curve depicting overall survival comparing patients with and without an increase in vascularity intensity of at least 10 a.u. from baseline to 12 weeks post-implantation.

There was no correlation between intensity gain from pre-implantation to 12 weeks and a reduction in the longest primary tumour size (R = 0.026; *p* = 0.927). Estimated mean survival did not differ between those with and without an increase in intensity of at least 10 a.u. at 12 weeks (15.64 vs. 21.37 months; *p* = 0.350) (Figure 6).

At 12 weeks post-implantation, the local disease control rate was 100%, but 3/20 patients (15%) had distant progressive disease with new metastases.

Over a median follow-up period of 11.2 months (IQR 7.8–12.8), 15/20 (75%) remained alive (estimated mean survival time of 17.9 months) with 3/20 (15%) demonstrating both local and distant disease progression, and 3/20 (15%) demonstrating distant progression only. The patients with only distant progression demonstrated stable local disease on imaging.

### 3.3. Chemotherapy

The median number of initial cycles of FOLFIRINOX was eight (IQR 8–11). Second-line chemotherapy was administered to six patients (30%) after 12 weeks due to poor tolerability (n = 3) and progressive disease (n = 3) with the first-line regimen.

### 3.4. GI Toxicity

Endoscopic assessment at 12 weeks revealed gastric antral erosions in 2/15 patients (13%); both had grade 1 nausea and grade 1 pain, and one also had grade 1 diarrhoea. Both had negative *Helicobacter pylori* serology, and one was on long-term aspirin. These two patients had 3.2 mL and 2.3 mL of ^32^P implanted transgastrically and transduodenally, respectively. One other patient had grade 2 nausea/vomiting (6.7%) and grade 2 diarrhoea (6.7%) without any endoscopic abnormalities. No grade 3 or higher acute GI toxicity was observed.

### 3.5. Quality of Life

The EORTC QLQ-C30 scores pre-implantation and 12 weeks post-implantation are shown in Table 2. Clinically relevant improvements were noted for cognitive functioning (*p* = 0.031), appetite loss (*p* = 0.027) and constipation (*p* = 0.039).

## 4. Discussion

The tumour microenvironment can influence the growth and progression of tumours, as well as treatment response. In PDAC, reduced vascularisation and hypoperfusion, in addition to a dense fibrous stroma, can compromise drug delivery to the tumour, which may contribute to a poor response to chemotherapy. No clinically available agent, thus far, has been shown in human trials to alter the microenvironment or result in vascular normalisation of PDAC. This study is the first to demonstrate that intra-tumoural ^32^P injection is associated with an increase in microvascularity. This potential has substantial implications for the management of PDAC.

This study compared tumour vascularity before ^32^P implantation after patients had already received two or three cycles of FOLFIRINOX, with vascularity at 4 and 12 weeks post-^32^P implantation, whilst chemotherapy continued. Although there are no data on the effects of FOLFIRINOX on pancreatic tumour vascularity, chemotherapy itself would not be expected to increase vascularity. Gemcitabine and nab-paclitaxel have been shown not to affect pancreatic tumour vascularity at 8 weeks post-chemotherapy commencement [23]. In one study with CE-EUS performed before and after gemcitabine, gemcitabine and cisplatin, and gemcitabine and radiotherapy (50–60 Gy), only 35% of patients had an increase in vascularity over a mean of 10.2 cycles, although, notably, increased intra-tumour blood flow was most commonly seen following two or three cycles of chemotherapy [7].

Although the mechanism responsible for the increased vascularity with combination ^32^P and FOLFIRINOX is unclear, the result may be that chemotherapy is delivered more effectively to cancer cells, thereby optimising its cytotoxicity. The reported effects of radiotherapy on vascularity and vessel density vary substantially [24,25,26]. In the majority of studies, low-dose radiotherapy increases perfusion in the early period but reduces blood flow thereafter, whilst high-dose radiotherapy results in severe vascular damage, but vascularity can recover by approximately 11 days to 3 weeks post irradiation [24,25,26,27,28]. Currently available radiotherapy options in PDAC include (a) conventional fractionated EBRT with 3D conformal radiation therapy (3DCRT) with small doses over a few weeks (50–56 Gy) and intensity-modulated radiation therapy (IMRT) defined by small doses (total 50–54 Gy) also over a few weeks (25 fractions) but with more precise spatial targeting and (b) hypofractionated radiotherapy with SBRT incorporating higher doses (total 40–60 Gy) over 1–5 visits with more focal delivery [29]. Although the total dose of ^32^P brachytherapy (100 Gy) is much higher than that given in conventional radiotherapy and SBRT, the rate of delivery, which extends over 81 days, is low [18,30]. This may have allowed for the increase in tumour vascularity observed in the present study. In contrast to EBRT and SBRT, which have not demonstrated an impact on survival, the outcomes of ^32^P combination therapy in LAPC have been encouraging [19,31]. This study demonstrated a 25% downstaging and a 100% local disease control rate at 12 weeks post-implantation. The role of increased vascularity in improving these clinical outcomes needs to be elucidated. The timing of brachytherapy within the course of chemotherapy in LAPC may be an important factor since increased vascularity post-^32^P implantation hypothetically suggests that chemotherapy will be more effective in the post-implantation phase. This suggests that ^32^P implantation should occur early during chemotherapy, in contrast to high-dose radiotherapy, which is traditionally given at the end of chemotherapy treatment. Assessing the longer-term impact of ^32^P implantation on tumour vascularity will help establish the optimal window for chemotherapy delivery.

The increased potential for metastases is one concern with increased microvessel density; however, this has been shown to be related to tumour-induced angiogenesis and not vascular normalisation [10,11]. The rate of distant metastases in this cohort was 30% over an 11.2-month median follow-up, which compares favourably with historical data for patients with LAPC, ranging from 44–78% within 1 year in patients treated with chemotherapy alone or chemoradiation [32,33,34,35]. This, in addition to good local tumour response, is consistent with the proposal that a combination of EUS-guided ^32^P microparticle implantation and chemotherapy increases vascular normalisation and is less likely to result in tumour-induced angiogenesis. Furthermore, the combination with chemotherapy may have a role in controlling the metastasis rate. This highlights the importance of a combination treatment compared to brachytherapy alone.

This study provided a unique opportunity for the endoscopic assessment of the gastrointestinal (GI) adverse effects of radiotherapy. Radiation-induced gastric injury, related to the cumulative radiation dose, is a common complication of radiotherapy for abdominal tumours [36]. Variable rates of GI toxicity post-radiotherapy have been described; one study demonstrated a 16.2% rate of grade 2 or higher gastrointestinal toxicity after single-fraction SBRT (25 Gy) and 7.8% after multi-fraction SBRT (median dose: 33 Gy) [37]. Other studies have shown 3.2–14% rates of grade 3 or higher GI toxicity post-SBRT, including GI bleeding, perforation and death [30,33,38]. The rates of GI toxicity post-EBRT are higher, with acute GI toxicity of grade 2 or higher occurring in 40% with 3DCRT (54 Gy/30 Fr) and 19% in those with IMRT (48 Gy/15 Fr). Only grades 1 and 2 GI toxicities were observed in our cohort, likely due to the rapid dose fall-off with increasing distance from the ^32^P brachytherapy source. The maximum penetration of the beta particles is 8.2 mm, which minimises the potential for damage to adjacent healthy tissue [39]. We observed gastric antral erosions in only two patients post-^32^P implantation. Whilst one patient had ^32^P implanted transgastrically, the other patient had implantation via a duodenal route, which should be less likely to result in gastric antral erosions. Even though the volumes of ^32^P implanted in these two patients were substantially higher than the median, other causes for erosions may have included aspirin or FOLFIRINOX chemotherapy. The true incidence of minor GI damage, such as mucosal erosions post-SBRT or EBRT, is unknown since the majority of these patients never undergo endoscopic assessment. However, given that the reported rates of grade 2 or higher GI toxicity post-SBRT or EBRT are substantial (16–40%), it appears likely that the rate of gastric erosions would be much higher than 13%.

Although increased tumour vascularity may allow better chemotherapy delivery and, therefore, improve chemotherapy effects, this study did not demonstrate any relationship between intensity gain following ^32^P implantation and tumour size reduction. This could be attributed to the small sample size. Alternatively, it could be that a certain threshold increase in perfusion is required to achieve maximal chemotherapeutic effect, after which further increases in vascularity provide no additional therapeutic benefit. In a study with 35% of patients demonstrating increased blood flow post-gemcitabine, an increase in blood flow correlated with a fall in CA19-9 levels and better clinical response [7]. Since a majority of patients in our study demonstrated an increase in blood flow, the ability to compare outcomes between those with increased flow and those without an increase was limited.

The concept that the combination of ^32^P implantation and chemotherapy can affect vascularity is novel and should be developed in further studies. Whilst we have demonstrated an increased flow of blood within tumour tissue after combination therapy, further mechanistic studies are worthwhile to determine whether this is vascular normalisation compared to tumour-induced angiogenesis.

The limitations of our study include its small sample size, which restricts the generalisability of the results. However, this study has raised the possibility that PDAC treatment can increase tumour vascularity, which should be explored further in larger studies. Another limitation is the lack of a control group, which would be required to determine whether changes in vascularity are attributable solely to ^32^P implantation or whether FOLFIRINOX has a role. This will need to be assessed in a further study; however, the current study is still the first to show a change in vascularity with any treatment for PDAC. Finally, being a pilot study, the assessment of changes was limited to 12 weeks post-implantation, given the burden on participants. Having shown promising results, examining vascular changes over a longer period would be worthwhile.

## 5. Conclusions

This is the first clinical study showing that an intervention can alter the vascularity of PDAC. The outcome may explain the encouraging outcomes of ^32^P implantation on local disease control, tumour downstaging and quality of life. More importantly, the current study has created a platform to better understand the potential benefits of chemoradiotherapy over chemotherapy alone in patients with LAPC. Larger comparative trials involving ^32^P implantation are warranted.

## Figures and Tables

**Table 1:** Baseline demographics and tumour characteristics of 20 patients.

Female Sex—No. (%)	11 (55%)
Median age (IQR)—years	65 (58–71)
ECOG performance-status score—no. (%) ^¥^	
0	9 (45%)
1	11 (55%)
Median Charlson Comorbidity Index Score (IQR)	5 (3–6)
Primary tumour stage—no. (%)	
T1	0 (0%)
T2	5 (25%)
T3	1 (5%)
T4	14 (70%)
Nodal status—no. (%)	
N0	17 (85%)
N1	3 (15%)
Pancreatic tumour location—no. (%)	
Head	7 (35%)
Neck	4 (20%)
Body	4 (20%)
Uncinate	5 (25%)
Target lesion longest diameter—median (IQR), mm	32 (27.5–38.75)
CA19.9—median (IQR); U/mL	121 (51–243)

^¥^ The Eastern Cooperative Oncology Group (ECOG) performance–status scores range from 0 to 5, with higher scores indicating greater disability.

**Table 2:** EORTC QLQ-C30 questionnaire results.

EORTC QLQ-C30 Scale	Median Score (/100) Pre-Implantation	Median Score (/100) 12 Weeks Post-Implantation	*p*-Value ^¥^
Global health status/QoL	50.0	58.3	0.036 *
Physical functioning	73.3	73.3	0.582
Role functioning	66.7	66.7	0.440
Emotional functioning	75.0	70.8	0.262
Cognitive functioning	66.7	83.3	0.031 *
Social functioning	58.3	66.7	0.345
Fatigue	44.4	38.9	0.138
Nausea and vomiting	16.7	0	0.439
Pain	33.3	16.7	0.152
Dyspnoea	33.3	0.0	0.492
Insomnia	33.3	33.3	0.739
Appetite loss	33.3	0.0	0.027 *
Constipation	16.7	0.0	0.039 *
Diarrhoea	33.3	33.3	0.230
Financial difficulties	0.0	0.0	0.236

^¥^ The *p*-value of the matched variables determined using the Wilcoxon signed-rank test.

## Data Availability

The raw data supporting the conclusions of this article will be made available by the authors on request.
